# Motivations for user satisfaction of mobile fitness applications: An analysis of user experience based on online review comments

**DOI:** 10.1057/s41599-022-01452-6

**Published:** 2023-01-03

**Authors:** Hyeongjin Ahn, Eunil Park

**Affiliations:** 1 Department of Applied Artificial Intelligence, Sungkyunkwan University, Seoul, Korea; 2 Department of Human–Artificial Intelligence Interaction, Sungkyunkwan University, Seoul, Korea

**Keywords:** Information systems and information technology, Business and management

## Abstract

Considering that mobile fitness applications are one of the necessities in our lives, the user perspective toward the application is a prominent research topic in both academia and industry with the goal of improving such services. Thus, this study applies two different natural language processing approaches, bag-of-words, and sentiment analysis, to online review comments of the applications to examine the effects of user experience elements. The review dataset collected from 16,461 users, after pre-processing, revealed the notable roles of perceived affection and hedonic values in determining user satisfaction with the application, whereas the effect of user burden on satisfaction was marginal. Several implications, as well as limitations of the study, were examined incorporating the findings with the statistical results.

## Introduction

Health and well-being are the most critical topics in our society as they affect an individual’s emotional and physical state (Meredith, 1988). With the rapidly increasing use of smartphones and wearable devices in the global healthcare industry, digital health has become one of the mainstreams of next-generation health and well-being.

In addition, because of the COVID-19 global pandemic, digital tools are being consistently examined to manage users’ fitness and health. To effectively respond to COVID-19 issues and maintain a healthy lifestyle, digital healthcare services are considered an effective alternative (Webster, 2020).

With this background, a number of new innovative technologies in mobile fitness applications have been examined in relation to user behavior to track general activity and physical conditions. In particular, applications have been introduced for monitoring weight loss and exercise (Schumer et al., 2018). In 2021, approximately 17 million users actively used mobile fitness applications (Statista, 2022a). The global fitness application industry accelerated with 40.61% growth in revenue in 2020, and approximately 14.64 billion USD in annual revenue is estimated by 2027 (Globenewswire, 2020; RunRepeat, 2021). With this trend, service providers have introduced several mobile fitness applications, with more than 30,000 health and fitness applications available globally (Statista, 2022b).

Considering the dissemination of mobile fitness applications as a tool to promote healthy lifestyle behavior (Oyibo et al., 2019), several scholars have investigated the behavior of users in adopting mobile fitness applications to acquire a better understanding of their perspective. Dhiman et al. (2019), for instance, employed the extended unified theory of acceptance and use of technology (UTAUT2) to explore the potential motivations influencing consumer acceptance of smartphone fitness applications. The structural results of a user survey confirmed that users’ personal innovativeness, habits, price value, social influence, self-efficacy, and effort expectancy had a notable impact on their behavioral intention to adopt the applications. In addition, Cai et al. (2022) integrated three information system theories: expectation-confirmation theory (ECT), the technology acceptance model (TAM), and the post-acceptance model of information systems continuance (PAM-ISC) to examine the user perspective toward fitness applications.

However, most prior research has focused on user-survey-oriented approaches. Thus, it is necessary to examine users’ perspectives on mobile fitness applications by considering other innovative approaches. With the emergence of online platforms for mobile application markets, online review data containing user opinions on specific mobile services are readily accessible and available (Hedegaard and Simonsen, 2013). Although several scholars have explored the user’s perception of adopting mobile fitness applications based on several information-oriented theories, only a few studies have explored the user experience (UX) of the applications through online review comments or the relationship between UX elements and user satisfaction levels.

To this end, this study employs two different natural language processing approaches to examine the effects of UX dimensions presented in online review comments on mobile fitness applications. First, a dictionary-based word count model is employed to measure the levels of perceived usability, usefulness, and affection in the comments. Second, a research model based on the sentimental dimensions in linguistic inquiry and word count (LIWC) is examined to explore the determinants of users’ perceived satisfaction with mobile fitness applications (Kim and Park, 2019). The following research questions (RQs) are presented.

- **RQ1**: What factors determine users’ satisfaction with mobile fitness applications?
- **RQ2**: Can users’ satisfaction be examined using the UX elements in their online review comments?

## Literature review

### User experience in mobile applications

When innovative services or products are proposed and introduced, perceived satisfaction is a determinant in their adoption. Considering the findings of prior research on user satisfaction, UX was utilized and employed as one of the key factors that significantly affect users’ perceived satisfaction (Garrett, 2010).

One of the widely employed definitions of UX is “*a person’s* *perceptions and responses that result from the use or anticipated use of a product, system or service*” (ISO 9241-210, 2010). In other words, the concept of UX focuses on individuals’ feelings or perceptions regarding certain services during the utilization process. On this basis, several scholars have explored the core components of UX and their influence on users’ satisfaction levels as well as their impact on the adoption prospect of the services (Park, 2019). Among these components, three notable elements—usability, usefulness, and affection—are emphasized as notable predictors in determining the user experience of mobile applications (Jang and Yi, 2017; Jang and Park, 2022).

With this trend, some notable academic foundations have attempted to propose and introduce definitions for usability, usefulness, and affection. Perceived usability is defined as “*the extent to which a particular service can enable users to achieve certain goals with ease, effectiveness and efficiency*” (Bevan, 1995). Perceived usefulness is defined as “*the degree to which an individual believes that using a particular system would enhance his or her overall job performance*” (Zhao et al., 2018), while perceived affection is defined as “*a user’s psychological response to the perceptual design details of the product or service*” (Demirbilek and Sener, 2003).

In information-oriented research, UX has been one of the fundamental concepts in investigating users’ perspectives toward specific information services or products. For example, Huang et al. (2019) attempted to examine the motivational factors determining the behavioral intention to use mobile reservation services through the TAM. The structural results indicated that users’ perceived usefulness and ease of use positively influenced their overall experiences and had a significant impact on their intention to use the service. In another example, Park (2020) introduced ECT and TAM to explore user satisfaction with smart wearable devices with respect to UX. Confirmatory factor analysis and structural equation modeling were conducted, and the results showed that usefulness, ease of use, enjoyment, and service/system quality had notable effects on satisfaction, which in turn affected the intention to use. Zardari (2021) investigated the determinants of the intention to use an e-learning by integrating a UX-based e-learning acceptance framework with TAM. Based on the responses collected from 650 participants, the results validated the significant roles of perceived ease of use, usefulness, information quality, self-efficacy, social influence, and benefits in determining user acceptance behavior of e-learning portal services.

Although several empirical studies have suggested notable implications for the concept of UX in addressing user behavior in mobile applications, only a few prior studies have focused on the user perspective toward fitness services in mobile environments. Therefore, the aim of this study is to investigate the determinants of perceived satisfaction for mobile fitness applications from the UX perspective.

### Review analysis

To acquire a better understanding of UX in mobile services, user review comments are a valuable resource for shedding light on users’ perspectives toward the services (Wahyono et al., 2017). In other words, analyzing review comments allows researchers in industry and academia to determine users’ intrinsic feelings, desires, and encountered UX issues.

Google Play Store, one of the global mobile application markets, allows users to share their opinions on the applications. Review comments, which are presented in the store, consist of the review date, title, satisfaction level (1–5), and comments on the application. The convenience of having access to the collected comments has prompted several scholars to examine users’ overall feelings and opinions on mobile applications through the online review comments in the store. Bae et al. (2021) examined user review comments on Airbnb, a shared house application, to compare the experiences of users with Western and Eastern cultural backgrounds (e.g., the United States and Hong Kong). Based on the review data collected from 32,867 users, the results indicated that both perceived affection and usefulness were determinants of the users’ satisfaction with the application. Kusuma et al. (2021) attempted to explore the antecedents of perceived satisfaction with a ferry e-ticket service by examining Apple App Store and Google Play Store reviews. The results from 358 respondents indicated that users’ perceived satisfaction was significantly related to their intrinsic values and purchasing behavior. In this context, online review comments are effective and convenient resources with abundant accessibility and availability for collecting honest responses from service users to assess overall user satisfaction.

Thus, based on the findings of prior research, this study utilizes online review comments as a significant resource for examining the UX of mobile fitness applications as well as potential determinants of user satisfaction.

### Gamification of mobile fitness applications

When a new service is introduced, user satisfaction can be determined based on the experience and perceptions of the user (Deng et al., 2010). On this basis, online services have applied various techniques to improve UX and extend user satisfaction to promote the adoption of the service. Among these, gamification has become one of the most frequently employed techniques for online services (Deterding et al., 2011). In general, gamification is defined as “*the application of game mechanisms and elements in a non-game context to enhance user experience*” (Huotari and Hamari, 2012). In other words, some notable game element characteristics, including challenges, competition, and badges, are utilized in the context of education, finance, and healthcare, among others.

Prior research has indicated that applying the concept of gamification to mobile applications can contribute to enhancing UX, thereby improving users’ continual intention to use the application.

For instance, Lin et al. (2017) introduced TAM to investigate users’ satisfaction with gamified interactive learning systems for special education children with developmental delay. Based on the data collected from 150 participants, the structural results validated that perceived ease of use, perceived usefulness, and perceived playfulness had notable effects on users’ satisfaction, which in turn affected their intention to use the service.

As another example, Wong et al. (2021) attempted to examine silver-generation users’ intention to adopt gamified mobile payment technologies and demonstrated that perceived enjoyment has notable effects on perceived game effectiveness, which in turn contributes to perceived game usefulness and forming a positive attitude toward the service. Based on these findings, this study examines the UX of gamified fitness applications as an indicator of user satisfaction.

## Study 1: Bag-of-words

### Data collection

Initially, the datasets of the top five mobile fitness applications in the Google US Play Store were collected. The keyword “fitness” was entered to narrow the search to mobile fitness applications, thereby selecting five popular applications. Reviews and satisfaction levels were collected from 15,000 users for the top five applications.

To further select the top five fitness applications with gamification, the same keyword “fitness” was used for the Google US Play Store, and the mobile fitness applications that met the gamification criteria proposed by prior gamification research (e.g., narrative or plots) (Neupane et al., 2020) were selected. Finally, 12,824 reviews and satisfaction levels for gamified mobile fitness applications were collected. All collected datasets are open at https://github.com/dxlabskku/Fitness_UX.

### Data pre-processing and our approach

Several natural language processing techniques including topic analysis, clustering, and bag-of-words are used to address UX components. Based on the findings of previous research on UX that bag-of-words can extract users’ preferences or requests expressed from online user reviews (Al-Ramahi and Noteboom, 2020), this study employs bag-of-words for quantitatively extracting UX components from the reviews (He and Deng, 2018). Each bag-of-words consisted of three variables, usability, usefulness, and affection, based on prior validated research (Jang and Park, 2022).

**Fig. 1:**
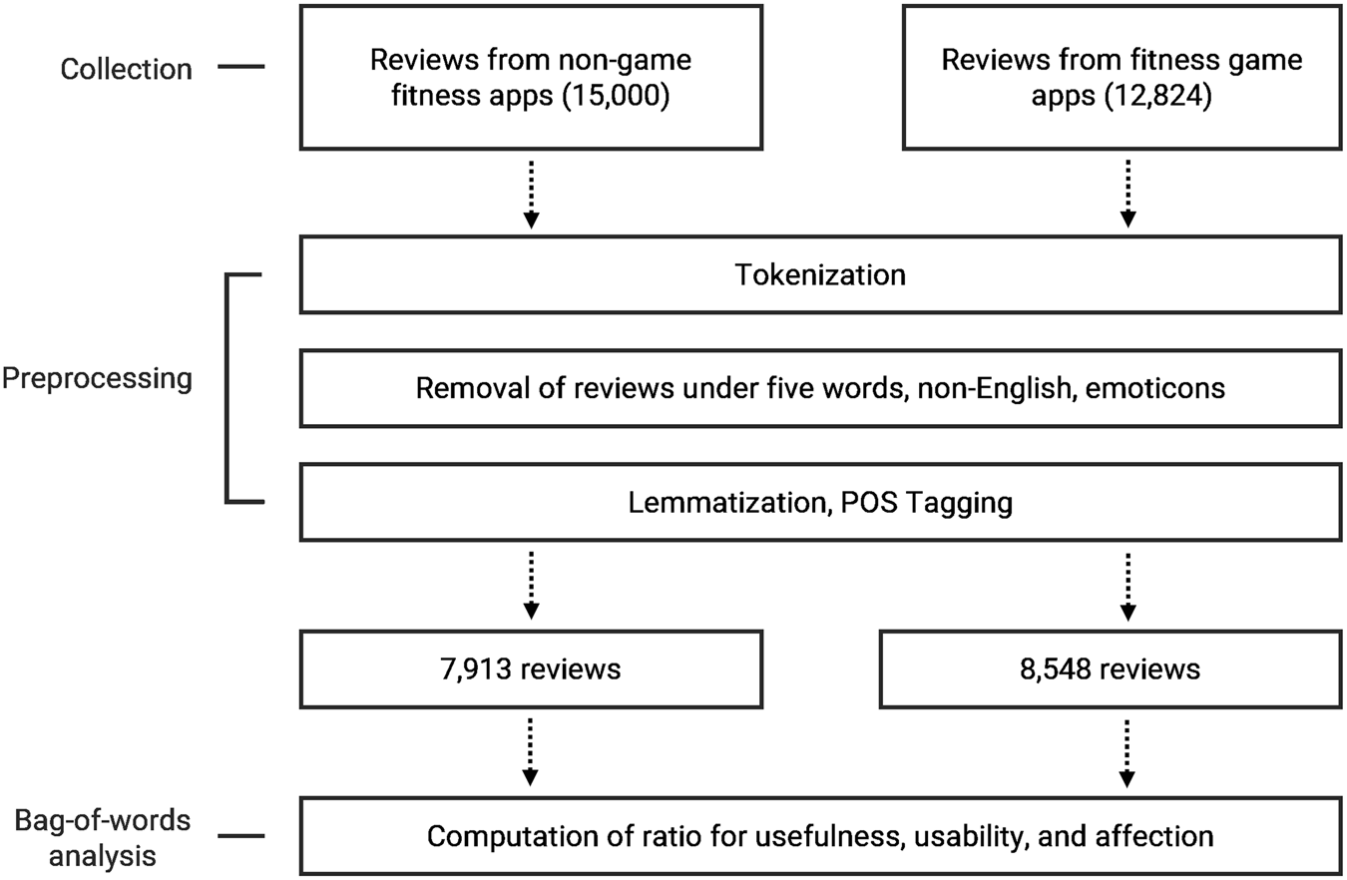
Data pre-processing procedures on the collected reviews from Google Play Store.

To measure the levels of perceived usability, usefulness, and affection revealed in user review comments, several text-processing steps were performed on the collected review comments to transform them into structural data (Fig. 1). Comments containing fewer than five words, emojis, emoticons, and non-English words, were removed. Both tokenization and lemmatization procedures were conducted. The proportion of each component in each word bag was computed to identify the number of words corresponding to each component in the review comments. For example, if a specific review with 10 words contained 3 words in the affection bag, the level of affection was calculated by dividing the value 3 by the total number of words in the review (3/10).

### Results

**Fig. 2:**
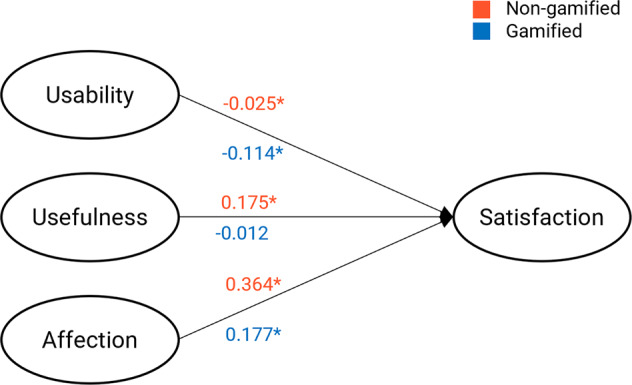
Summary of the results in Study 1 (*β*); *p-value is lower than 0.05.

Multiple regression analysis was used to investigate the effects of the UX elements in online reviews. Figure 2 summarizes the results. In the case of non-gamified mobile fitness applications (*R*^2^ = 0.158), all factors had notable effects on satisfaction. Affection (*M* = 11.25%, SD = 13.83%; *β* = 0.364, *p* < 0.001) had a significant effect on satisfaction; similarly, usefulness (*M* = 5.75%, SD = 10.68%; *β* = 0.175, *p* < 0.001) and usability (*M* = 5.21%, SD = 9.05%; *β* = −0.025, *p* < 0.05) were also related to satisfaction.

In the case of gamified mobile fitness applications (*R*^2^ = 0.050), no notable effects of usefulness (*M* = 1.92%, SD = 5.53%; *β* = −0.012, *p* = 0.255) were observed, unlike the results of non-gamified fitness applications. Moreover, two elements, namely usability (*M* = 3.65%, SD = 7.22%; *β* = −0.114, *p* < 0.001) and affection (*M* = 9.50%, SD = 12.73%; *β* = 0.177, *p* < 0.001), had an effect on satisfaction.

## Study 2: Sentiment analysis

### Data collection and pre-processing

The same dataset described in the section “Data collection” (Study 1) was employed for sentiment analysis.

### Our approach and hypotheses

To establish a link between sentiment analysis and UX dimensions, several prior studies proposed novel text analysis/mining approaches (Park, 2019). As a representative example, Jang and Yi (2017) investigated user satisfaction by extracting UX elements from online reviews of mobile devices. Considering 4380 online reviews, they computed several key elements (e.g., hedonic values and user burden) related to UX dimensions. The statistical results indicated that there were significant relationships between the UX dimensions extracted by sentiment analysis and user satisfaction.

In addition, Park (2019) extracted UX elements from online review comments on airline services using LIWC. Using more than 100,000 samples, they extracted crucial elements of UX—hedonic values, user burden, expectation confirmation, and pragmatic values—with social values, and further validated that these elements were significant determinants of user satisfaction with the services.

Thus, based on the findings of prior research on sentiment analysis and UX dimensions, sentiment analysis was adopted in this study as one of the main techniques for determining the link between online review comments and UX dimensions. A summary is presented in Table 1 based on previously validated research (Jang and Yi, 2017; Jang and Park, 2022).

**Table 1:** Summary of the measurements and corresponding LIWC categories.

Constructs	LIWC category	Scale
Hedonic values	Positive emotion	0–1.0
User burden values	Negative emotion	0–1.0
Expectation-confirmation	Comparisons	0–1.0
Pragmatic values	Work, leisure, and home	0–1.0
Social values	Social words	0–1.0
Satisfaction	–	Ratings (1: extremely unsatisfied–5: extremely satisfied)

Considering the summary presented in Table 1, the following hypotheses are proposed:

- **H1**. Hedonic values have a significant effect on perceived satisfaction.
- **H2**. User burden values have a significant effect on satisfaction.
- **H3**. Expectation confirmation has a significant effect on perceived satisfaction.
- **H4**. Pragmatic values have a significant effect on perceived satisfaction.
- **H5**. Social values have a significant effect on perceived satisfaction.

#### Research model

**Fig. 3:**
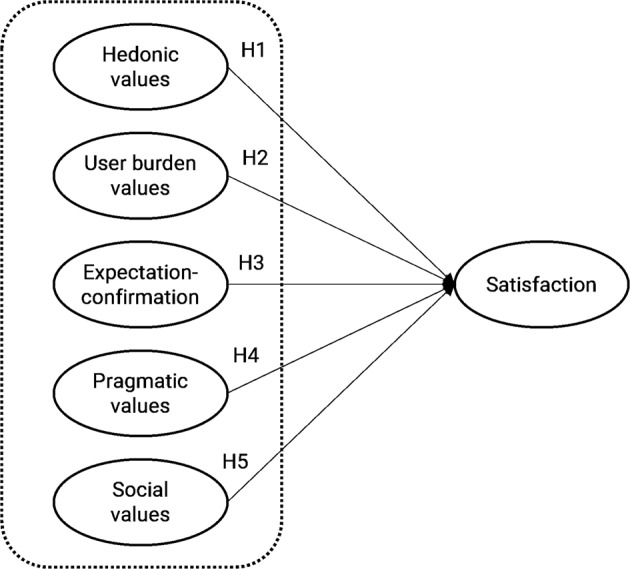
Research model in Study 2.

Figure 3 illustrates the research model based on the proposed hypotheses. The research model determined that hedonic values, user burden values, expectation confirmation, and pragmatic and social values are potential determinants of users’ satisfaction with mobile fitness applications.

### Results

Tables 2 and 3 summarize the results of multiple linear regression analysis considering both non-gamification and gamification.

**Table 2:** Summary of the results for non-gamified fitness applications.

Hypothesis	Standard coefficient	Critical ratio	*p*-value	Results
H1. Hedonic values → Satisfaction	0.316	40.579	<0.001	Supported
H2. User burden values → Satisfaction	−0.162	−20.917	<0.001	Supported
H3. Expectation-confirmation → Satisfaction	0.004	0.004	0.528	Not supported
H4. Pragmatic values → Satisfaction	0.030	3.911	<0.001	Supported
H5. Social values → Satisfaction	0.086	11.154	<0.001	Supported

**Table 3:** Summary of the results for gamified fitness applications.

Hypothesis	Standard coefficient	Critical ratio	*p*-value	Results
H1. Hedonic values → Satisfaction	0.278	31.738	<0.001	Supported
H2. User burden values → Satisfaction	−0.220	−26.347	<0.001	Supported
H3. Expectation-confirmation → Satisfaction	0.033	3.945	<0.001	Supported
H4. Pragmatic values → Satisfaction	0.019	2.149	<0.05	Supported
H5. Social values → Satisfaction	0.112	13.278	<0.001	Supported

According to the results for the collected review data on non-gamified fitness applications, user satisfaction (*R*^2^ = 0.149) was significantly influenced by four constructs: hedonic values (H1, *β* = 0.316, CR = 40.579, *p* < 0.001), user burden values (H2, *β* = −0.162, CR = −20.917, *p* < 0.001), pragmatic values (H4, *β* = 0.030, CR = 3.911, *p* < 0.001), and social values (H5, *β* = 0.086, CR = 11.154, *p* < 0.001), whereas the relationship between expectation confirmation and satisfaction was not significant (H3, *β* = 0.004, CR = 0.528, *p* = 0.597).

In gamified fitness applications, user satisfaction (*R*^2^ = 0.164) was significantly influenced by all five constructs: hedonic values (H1, *β* = 0.278, CR = 31.738, *p* < 0.001), user burden values (H2, *β* = −0.220, CR = −26.347, *p* < 0.001), expectation confirmation (H3, *β* = 0.033, CR = 3.945, *p* < 0.001), pragmatic values (H4, *β* = 0.019, CR = 2.149, *p* < 0.05), and social values (H5, *β* = 0.112, CR = 13.278, *p* < 0.001).

### Additional approaches

In addition to the two employed approaches, bag-of-words and sentiment analysis, other widely used methodologies, both latent Dirichlet allocation (LDA) topic analysis, and *K*-Means clustering were considered to explore if there might be better natural language processing methods for examining the UX of mobile fitness applications.

To identify UX dimensions from online reviews, LDA topic analysis was used. LDA is an unsupervised machine learning technique, which extracts implicit topics from reviews and captures the keywords corresponding to each topic (Ali et al., 2021). For example, Yoon et al. (2022) attempted to explore the impact of dimensions of UX on user satisfaction with smart speakers by employing LDA topic analysis. Based on the collected data from 46,715 user reviews, the results validated the relationship between UX dimensions and user satisfaction.

This study also employs a *K*-Means clustering analysis for capturing UX dimensions. It is one of the simplest algorithm techniques for extracting a specific number of clusters denoted as *k* and defining each cluster as homogeneous keyword sets from online reviews (Fry and Manna, 2016). Santoso and Schrepp (2019) utilized a *K*-Means clustering analysis to extract UX aspects, based on the semantic similarity of various product categories with different cultural backgrounds. The results confirmed that the effects of product categories on the importance of UX dimensions are greater than those of cultural differences, based on the collected responses from 114 participants.

Moreover, the multiple linear regression results of two additional approaches were suitable for identifying the UX dimensions of mobile fitness applications. The results of the LDA topic analysis showed the weakness of explanatory satisfaction power in both non-gamified (*R*^2^ = 0.046) and gamified (*R*^2^ = 0.074) fitness applications. In the case of *K*-Means clustering analysis, the results on both non-gamification (*R*^2^ = 0.111) and gamification fitness applications (*R*^2^ = 0.053) also did not provide great explanatory power of user satisfaction.

## Discussion and conclusion

This study investigated the effects of UX elements of mobile fitness applications on user satisfaction. To quantitatively extract the elements, two text-processing approaches were employed. The concept of gamification issues in mobile fitness applications was also considered.

Based on the dataset of 16,461 online review comments, the results of two multiple regression analyses were presented. First, using the bag-of-words approach, three UX elements, namely usability, usefulness, and affection, were extracted from the comments and quantitatively examined. Usability and affection were identified as key determinants of user satisfaction in both gamified and non-gamified applications. Although usefulness was significantly related to satisfaction in non-gamified applications, it had no effect on satisfaction in gamified applications. Moreover, users of non-gamified applications were more likely to express greater levels of usability and affection than those of gamified applications. It implies that users’ affective experience in using mobile fitness applications is particularly crucial to improve their perceived satisfaction.

Considering the sentiment analysis approach, five elements of UX—hedonic values, user burden values, expectation confirmation, pragmatic values, and social values—were extracted from online review comments. To explore the theoretical framework for understanding the relationship between UX elements and satisfaction, this study employed multiple linear regression analysis. The results showed that hedonic, user burden, pragmatic, and social values were key predictors of user satisfaction, whereas there was no notable relationship between expectation confirmation and satisfaction in non-gamified mobile fitness applications. In contrast, all elements were significantly related to satisfaction in gamified mobile fitness applications. This indicates that users’ perceived positive emotion is the most significant predictor of satisfaction in both non-gamified and gamified applications, whereas negative emotions are a major obstacle to obtaining satisfaction (Jang and Park, 2022).

Based on the findings of this study, several practical implications are presented. Considering that this study employed two approaches to extract UX factors from review comments, UX practitioners can utilize the suggested approaches to seek interventions to improve user satisfaction based on UX. Second, because both users’ perceived affection and usability levels were examined as key direct predictors of their satisfaction, practical application developers should pay more attention to improving the levels in non-gamified applications. Lastly, consistent with the findings of several prior studies on customers’ emotional state and their satisfaction (Berrouiguet et al., 2016; Xiaofei et al., 2021), service providers should consider users’ affective and emotional values to improve their satisfaction by analyzing their current services.

Although several findings and implications have been presented, certain limitations remain to be addressed. First, user demographic information was not included in the study. As presented in several prior user-oriented studies, users’ socio-demographic information can have notable effects on their satisfaction with specific mobile applications (Lee and Han, 2015). Second, because only the Google US Play Store was considered, the results may be difficult to generalize. Considering these limitations, future studies can extend the findings of this study to provide a better understanding of the role of UX in mobile applications. Lastly, applying more recent and complex natural language processing techniques can deliver better insights and results in investigating user experience (e.g. machine and deep learning techniques (Aslam et al., 2020, Sadiq et al., 2021). Thus, future research can address these limitations based on the findings of the current study.

## Data Availability

We publicly open our collected dataset at https://github.com/dxlabskku/Fitness_UX.

## References

[CR1] Al-Ramahi M, Noteboom C (2020). Mining user-generated content of mobile patient portal: dimensions of user experience. ACM Trans Soc Comput.

[CR2] Ali T, Marc B, Omar B, Soulaimane K (2021). Exploring destinationas negative e-reputation using aspect based sentiment analysis approach: case of Marrakech destination on tripadvisor. Tour Manag Perspect.

[CR3] Aslam N, Ramay WY, Xia K (2020). Convolutional neural network based classification of app reviews. IEEE Access.

[CR4] Bae K, Jang Y, Han J, Park E, del Pobil AP (2021) A quantitative analysis of satisfaction on Airbnb from UX perspectives: the comparison between the United States and Hong Kong. In: Lee S, Hanzo L, Ismail R (eds) Proceedings of the IMCOM ’21. IEEE, pp. 1–4

[CR5] Berrouiguet S, Baca-García E, Brandt S (2016). Fundamentals for future mobile-health (mhealth): a systematic review of mobile phone and web-based text messaging in mental health. J Med Internet Res.

[CR6] Bevan N (1995). Measuring usability as quality of use. Softw Qual J.

[CR7] Cai J, Zhao Y, Sun J (2022). Factors influencing fitness app users’ behavior in china. Int J Hum–Comput Interact.

[CR8] Demirbilek O, Sener B (2003). Product design, semantics and emotional response. Ergonomics.

[CR9] Deng L, Turner DE, Gehling R (2010). User experience, satisfaction, and continual usage intention of it. Eur J Inf.

[CR10] Deterding S, Sicart M, Nacke L et al. (2011) Gamification. Using game-design elements in non-gaming contexts. In: Tan D, Begole B, Kellogg WA (eds) CHI’11 extended abstracts on human factors in computing systems. ACM, pp. 2425–2428

[CR11] Dhiman N, Arora N, Dogra N (2019). Consumer adoption of smartphone fitness apps: an extended UTAUT2 perspective. J Indian Bus Res.

[CR12] Fry C, Manna S (2016) Can we group similar amazon reviews: a case study with different clustering algorithms. In: Li T, Scherp A, Ostrowski D, Wang W (eds) Proceeding of the ICSC ’16. IEEE, pp. 374–377

[CR13] Garrett JJ (2010) The elements of user experience: user-centered design for the web and beyond. Pearson Education.

[CR14] Globenewswire (2020) Fitness App market to reach USD 14.64 billion by 2027. https://www.globenewswire.com/news-release/2020/05/14/2033925/0/en/Fitness-App-Market-To-Reach-USD-14-64-Billion-By-2027-Reports-and-Data.html. Accessed 6 May 2022

[CR15] He X, Deng L (2018) Deep learning in natural language generation from images. In: Deng L, Liu Y (eds) Deep learning in natural language processing. Springer, pp. 289–307

[CR16] Hedegaard S, Simonsen JG (2013) Extracting usability and user experience information from online user reviews. In: Mackay et al. (eds) Proceedings of the CHI ’13. ACM, pp. 2089–2098. 10.1145/2470654.2481286

[CR17] Huang YC, Chang LL, Yu CP (2019). Examining an extended technology acceptance model with experience construct on hotel consumers’ adoption of mobile applications. J Hosp Mark Manag.

[CR18] Huotari K, Hamari J (2012) Defining gamification: a service marketing perspective. In: Lugmayr A (ed) Proceedings of the 16th international academic MindTrek conference. ACM, pp. 17–22

[CR19] ISO 9241-210 (2010) Ergonomics of human–system interaction—Part 210: human-centered design for interactive systems (formerly known as 13407) https://www.iso.org/standard/52075.html

[CR20] Jang J, Yi MY (2017) Modeling user satisfaction from the extraction of user experience elements in online product reviews. In: Mark G, Fussell S, Lampe C et al (eds) Proceedings of CHI ’17. ACM, pp. 1718–1725

[CR21] Jang Y, Park E (2022). Satisfied or not: user experience of mobile augmented reality in using natural language processing techniques on review comments. Virtual Real.

[CR22] Kim J, Park E (2019). Beyond coolness: predicting the technology adoption of interactive wearable devices. J Retail Consum Serv.

[CR23] Kusuma DCC, Fauzi MI, Rifdi M et al. (2021) The determinant factors of user satisfaction on ferry e-ticket purchase (ferizy): integration of the utaut and is success model. In: Musu W (ed) Proceedings of the ICORIS ’21. IEEE, pp. 1–7

[CR24] Lee E, Han S (2015). Determinants of adoption of mobile health services. Online Inf Rev.

[CR25] Lin HC, Chiu YH, Chen YJ (2017). Continued use of an interactive computer game-based visual perception learning system in children with developmental delay. Int J Med Inform.

[CR26] Meredith MD (1988). Activity or fitness: Is the process or the product more important for public health?. Quest.

[CR27] Neupane A et al. (2020) A review of gamified fitness tracker apps and future directions. In: Mirza-Babaei P et al (eds) Proceedings of the annual symposium on computer–human interaction in play. ACM, pp. 522–533

[CR28] Oyibo K et al. (2019) Ben’fit: design, implementation and evaluation of a culture-tailored fitness app. In: Papadopoulos GA et al (eds) Adjunct publication of the 27th conference on user modeling, adaptation and personalization. ACM, pp. 161–166

[CR29] Park E (2019). Motivations for customer revisit behavior in online review comments: analyzing the role of user experience using big data approaches. J Retail Consum Serv.

[CR30] Park E (2020). User acceptance of smart wearable devices: an expectation-confirmation model approach. Telemat Inform.

[CR31] RunRepeat (2021) Fitness industry statistics 2021–2028 [market research]. https://runrepeat.com/fitness-industry. Accessed 6 May 2022

[CR32] Sadiq S, Umer M, Ullah S (2021). Discrepancy detection between actual user reviews and numeric ratings of google app store using deep learning. Expert Syst Appl.

[CR33] Santoso HB, Schrepp M (2019). The impact of culture and product on the subjective importance of user experience aspects. Heliyon.

[CR34] Schumer H, Amadi C, Joshi A (2018). Evaluating the dietary and nutritional apps in the google play store. Healthc Inform Res.

[CR35] Statista (2022a) Health and fitness apps—statistics & facts. https://www.statista.com/topics/9204/health-and-fitness-apps/. Accessed 6 May 2022

[CR36] Statista (2022b) Leading health and fitness apps in the U.S. 2018, by users. https://www.statista.com/statistics/650748/health-fitness-app-usage-usa/. Accessed 6 May 2022

[CR37] Wahyono T, Warnars HLHS, Wijaya BS et al. (2017) Building a popular mobile application by utilizing user feedback. In: Purnomo HD (ed) Proceedings of the ICITech ’17. IEEE, pp. 1–6

[CR38] Webster P (2020). Virtual health care in the era of covid-19. The Lancet.

[CR39] Wong D, Liu H, Meng-Lewis Y (2021). Gamified money: exploring the effectiveness of gamification in mobile payment adoption among the silver generation in china. Inf Technol People.

[CR40] Xiaofei Z, Guo X, Ho SY (2021). Effects of emotional attachment on mobile health-monitoring service usage: an affect transfer perspective. Inf Manag.

[CR41] Yoon SH, Park GY, Kim HW (2022). Unraveling the relationship between the dimensions of user experience and user satisfaction: a smart speaker case. Technol Soc.

[CR42] Zardari BA (2021). Development and validation of user experience-based e-learning acceptance model for sustainable higher education. Sustainability.

[CR43] Zhao Y, Ni Q, Zhou R (2018). What factors influence the mobile health service adoption? A meta-analysis and the moderating role of age. Int J Inf Manag.

